# Emotional intelligence as a mediator of anxiety and role ambiguity among nurses caring for critically ill geriatric patients

**DOI:** 10.1186/s12912-025-03437-7

**Published:** 2025-07-08

**Authors:** Asmaa Mahmoud Ali Ibrahim, Nadia Abdelnasser, Mohamoud Abdelwahab Khedr, Eman Mahmoud Mohammed Shoukr

**Affiliations:** 1 Gerontological Nursing Department, Faculty of Nursing, Alexandria University, Alexandria, Egypt; 2 Gerontological Nursing Department, Faculty of Nursing, South Valley University, South Valley, Egypt; 3 Psychiatric and Mental Health Nursing Department, Faculty of Nursing, Alexandria University, Alexandria, Egypt; 4 Community Health Nursing Department, College of Nursing, Jouf University, Sakaka, Saudi Arabia

**Keywords:** Emotional intelligence, Anxiety, Role ambiguity, Critical care nurses, Path analysis

## Abstract

**Background:**

Critical care nurses caring for critically ill geriatric patients face pressures, including anxiety and role ambiguity, impairing their ability to do their jobs. In critical care settings, geriatric patients present special challenges due to their complex medical issues, frailty, cognitive impairments (such as dementia or delirium), and age-related elevated risks.

**Aim:**

Investigate the role of emotional intelligence as a mediator between anxiety and role ambiguity among nurses caring for critically ill geriatric patients.

**Methods:**

A descriptive correlational analytical research design was followed. The study subjects were selected using the purposive sampling technique and involved 250 Intensive care units at South Valley University hospitals in Qena City, Egypt. Four tools were used for data collection: sociodemographic and occupational data of critical care nurses, the Wong and Law Emotional Intelligence Scale (WLEIS), the Generalized Anxiety Disorder 7-item (GAD-7), and Job Role ambiguity.

**Results:**

The study reported a significant negative relationship between ICU nurses’ emotional intelligence and anxiety level (*r*=-0.366, and *P* = 0.000), and a positive relationship between emotional intelligence and role ambiguity (*r*=-0.630, and *P* = 0.000), while a negative relationship was observed between anxiety and role ambiguity (*r*=-0.327, and *P* = 0.000).

**Conclusion:**

The study highlights the importance of emotional intelligence in managing anxiety and role ambiguity among nurses caring for critically ill geriatric patients. Fostering emotional intelligence can improve job satisfaction, job well-being, and patient care quality by clarifying role expectations.

**Clinical trial number:**

Not applicable.

## Introduction

The healthcare setting, particularly in intensive care units (ICUs), may be emotionally and psychologically stressful for nurses, especially when caring for geriatric patients who are in critical condition. Geriatric patients in critical care settings pose unique challenges because of their complicated medical problems, fragility, cognitive impairments (such as dementia or delirium), and the increased risks that come with age. In addition, end-of-life care, moral conundrums, and communicating with family members about the prognosis and care objectives sometimes provide emotional difficulties for nurses [1]. ICU nurses may experience severe stress due to these variables, which can negatively affect their well-being, job performance, and the standard of critically ill geriatric patient care by causing feelings of anxiety and role ambiguity [2, 3].

The Job Demands-Resources (JD-R) paradigm, which contends that personal resources, such as emotional intelligence (EI), assist in counteracting the impacts of job demands, such as role ambiguity, on psychological strain, is used to support the theoretical underpinnings of this study. Emotional intelligence (EI) serves as a psychological tool that helps nurses recognize, comprehend, and successfully handle emotional difficulties in high-stress settings such as essential geriatric care, which in turn lessens anxiety associated with job ambiguity. Thus, by placing the JD-R model inside a well-established occupational stress framework, it strengthens the conceptual contribution of this research and validates the suggested mediating function of EI [4, 5].

Globally, Anxiety disorders cost 2.08% of worldwide healthcare spending annually, making them a serious public health concern [6]. Nursing professionals, including ICU nurses, have anxiety disorders to differing degrees and in different nations [7]. According to a prospective longitudinal study, 50% of medical personnel, particularly critical care unit nurses, reported experiencing substantial levels of anxiety during the Italian COVID-19 pandemic [8]. In Oman, 34.8% of ICU nurses faced anxiety disorders, while in China [9], 22.3% of ICU nurses reported experiencing anxiety [10].

Anxiety among healthcare professionals, including ICU nurses, can manifest as a response to multiple reasons, such as the ongoing stress of caring for critically ill geriatric patients, dealing with high death rates, and navigating the uncertainty surrounding patients’ prognosis [11, 12]. Anxiety can contribute to decreased job satisfaction and burnout by affecting communication, emotional resilience, and cognitive functioning. Uncontrolled anxiety has been found to have a detrimental impact on nurses’ quality of life, ability to concentrate, and ability to make decisions [13–15]. The most prevalent and significant element influencing anxiety at work in nursing practice is role ambiguity [16]. Role ambiguity is characterized as a state in which nurses lack clarity regarding their job duties, expectations, and boundaries. This condition can cause confusion, stress, and reduced work effectiveness [17].

Role ambiguity in the workplace may result from the growing and more complicated duties that nurses currently perform. Uncertainty over nurses’ obligations has resulted from overlapping or contradictory tasks and responsibilities and inadequate communication within multidisciplinary healthcare teams [18, 19]. However, additional factors that contribute to ambiguity in ICU nurses’ roles include the absence of knowledge regarding managing critically ill geriatric patients’ diseases, the unclear needs of supervisors, and the possible expectations of coworkers [20]. Additionally, role ambiguity is positively correlated with anxiety, stress, and burnout, according to several research studies [21, 22]. Role ambiguity adversely affects nurses’ mental health because it hinders the efficient execution of tasks, leading to unpleasant feelings, including irritation and worry. Although role ambiguity and anxiety have been investigated previously, the relationship between role ambiguity and anxiety among critical care unit nurses has not received significant attention [20, 23].

Emotional Intelligence (EI), defined as the ability to identify, comprehend, control, and affect one’s own emotions and those of others, has become a viable defense against the detrimental effects of role ambiguity and anxiety [24]. EI is essential for ICU nurses caring for critically ill geriatric patients because it helps them better control their emotions, build stronger connections with others, communicate more effectively, cope with stress, and handle job demands. High emotional intelligence ICU nurses are more capable of demonstrating empathy, managing psychological and emotional difficulties, and making wise choices in emergencies [25]. Numerous researchers have found a strong correlation between emotional intelligence, mental health, and subjective well-being [26, 27]. According to a longitudinal study’s findings, emotional intelligence may help healthcare professionals feel less anxious during stressful situations, such as the COVID-19 pandemic [28]. Furthermore, emotional intelligence can decrease the negative effects of role ambiguity. Research conducted in Indonesia revealed that emotional intelligence minimized the influence of role ambiguity on employee burnout [29].

Whereas previous studies have addressed the impact of role ambiguity and anxiety among ICU nurses, the majority have focused on general nursing populations and ignored psychological mechanisms that may explain this relationship. In particular, the mediating role of EI has received limited attention, especially in the context of geriatric critical care, which presents unique emotional and ethical demands. This study fills this gap by examining Emotional Intelligence (EI) as a mediator between role ambiguity and anxiety among nurses caring for critically ill older adults, offering a novel contribution grounded in the Job Demands-Resources model [30, 31]. Additionally, there are some studies on anxiety and role ambiguity [1, 2]. Nevertheless, there is a dearth of studies on how role ambiguity and emotional intelligence affect anxiety in an intensive care unit. Thus, the current study aims to examine emotional intelligence as a mediator of anxiety and role ambiguity among nurses caring for critically ill geriatric patients.

## Methods

### Participants

A descriptive correlational analytical research design was used. The data was collected from Intensive Care Units at South Valley University (SVU) hospitals in Qena City, Egypt. Data collection began in early November and finished at the end of December 2024. South Valley University hospitals are the largest teaching hospitals in Qena city. They consist of three different buildings. The hospitals offer the following medical services: cardiology, neurology, urology, surgery, emergency, and trauma for Qena, Luxor, and Red Sea Governorate populations. These hospitals have the greatest daily attendance and total number of members in comparison to other hospitals in Qena City.

### Sample size

We estimated the required sample size for the study using G*Power Windows 3.1.9.7 software, applying parameters such as one group, two predictors, a significance level of 0.05 to control the probability of a Type I error, an effect size of 0.5, and a desired power of 0.95 to minimize the probability of a Type II error. The analysis determined a minimum sample size of 227 participants; however, we increased this to 250 during data collection to account for a 10% non-response rate. Nurses were selected based on specific inclusion criteria, which included being an intensive care unit nurse, working with older adults, having at least one year of professional experience, and being willing to participate in the study.

### Recruitment process

**Fig. 1:**
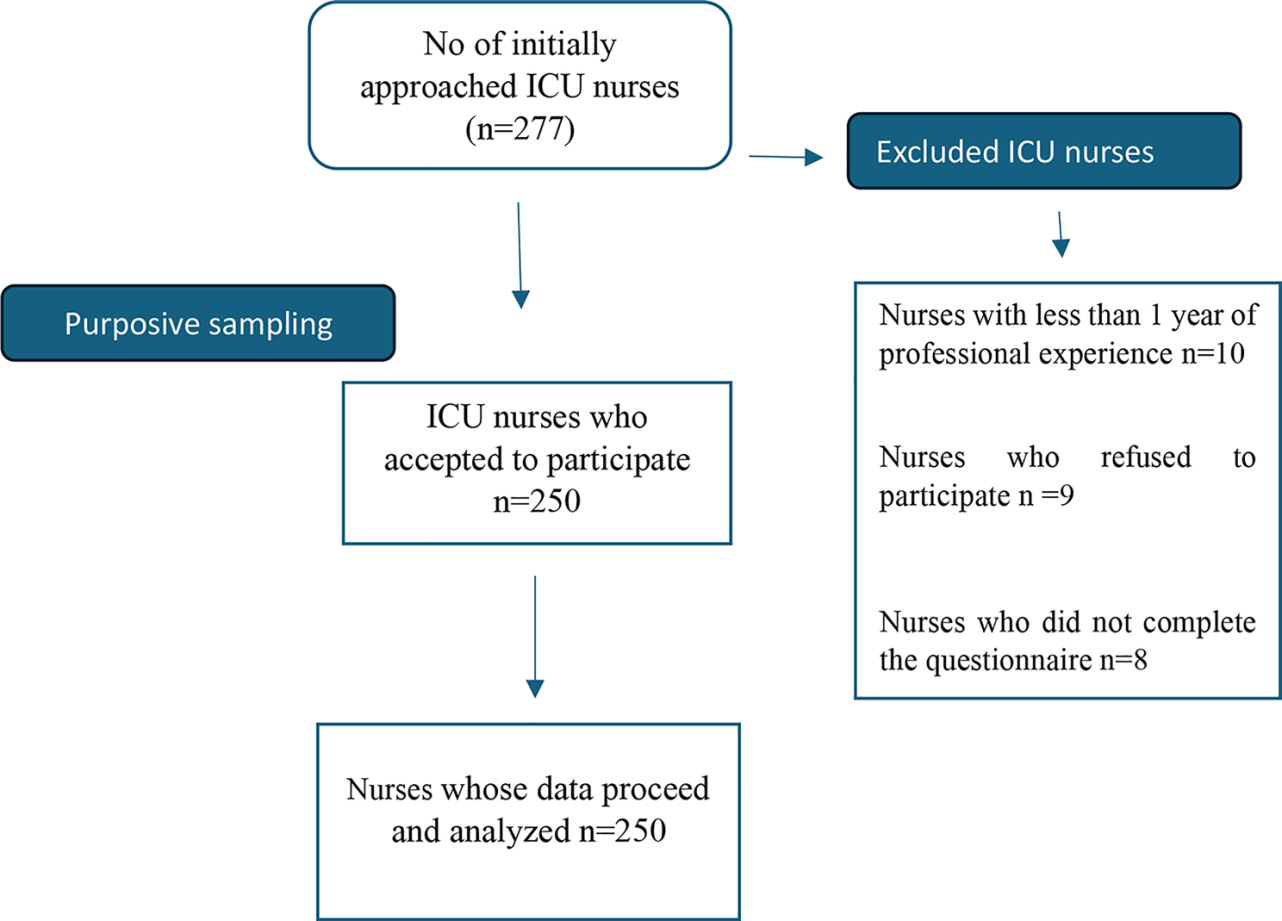
Flow chart for participants recruitment

The study invited approximately 277 ICU nurses to participate, but 10 were excluded due to having less than 1 year of professional experience. Additionally, nine nurses refused to participate, eight accepted but did not complete all the questionnaires and withdrew from the study **(**Fig. 1**)**. After the selection of nurses according to the inclusion criteria, the researcher formulated a questionnaire using the Google form and contacted participants using the WhatsApp application to send the questionnaire link. Google Forms responses are stored in a worksheet that can only be accessed through a Google account login.

### Study tools

To gather the required data, four instruments were used as follows:

#### Sociodemographic and occupational data of critical care nurses

The researchers developed it and included the following data about critical care nurses: age, sex, marital status, level of education, profession, and ICU experience.

#### Wong and law emotional intelligence scale (WLEIS)

Emotional Intelligence was measured by Wong and Law’s Emotional Intelligence Scale (WLEIS), a self-report measure developed by Law et al. 2004 [32]. It is a short 16-item measure of emotional intelligence. It consisted of four dimensions: [1] self-emotional appraisal (SEA) [2], others’ emotional appraisal (OEA) [3], use of emotion (UOE), and [4] regulation of emotion (ROE). It is a seven-point Likert scale, and its score ranged from strongly disagree = 1, disagree = 2, slightly disagree = 3, neither agree nor disagree = 4, strongly agree 5, agree = 6, and slightly agree = 7. The score ranged from 16 to 112. For each dimension, a score from four to twelve is considered mild, a score from 13 to 20 is considered moderate, and a score from 21 to 28 is considered high. As regards the total level of emotional intelligence, 16 to 48 are considered mild, 49 to 80 are considered moderate, and 81 to 122 are considered high levels of emotional intelligence. This scale had a Cronbach’s alpha coefficient of 0.862, indicating strong internal consistency.

#### Generalized anxiety disorder 7-item (GAD-7)

The GAD-7 was developed by Spitzer et al. (2006) [33], with an educational grant from Pfizer Inc. Scoring GAD-7 Anxiety Severity is calculated by assigning scores of 0, 1, 2, and 3 to the response categories, respectively, of “not at all,” “several days,” “more than half the days,” and “nearly every day.” GAD-7 total score for the seven items ranges from 0 to 21.

Minimal anxiety ranges from zero to four, mild anxiety ranges from five to nine, moderate anxiety ranges from 10 to 14, and severe anxiety ranges from 15 to 21. With an alpha of 0.885, the Arabic version of the GAD-7 showed good dependability.

#### Job role ambiguity

This tool, known as Job Role Ambiguity, conceptualized by Breaugh and Colihan (1994), encompasses nine items that assess role ambiguity across three distinct domains: [1] work methods, characterized as the lack of clarity that employees experience regarding the appropriate techniques to employ for job execution; [2] work scheduling, characterized as the uncertainty surrounding the order in which tasks should be completed, the distribution of time allocated to those tasks, and the sequence for undertaking specific activities; and [3] performance evaluation, characterized as the ambiguity employees face relating to the criteria utilized for evaluating and determining the adequacy of job performance [34]. Data collection is facilitated through a 7-point Likert-type scale, wherein a score of 1 corresponds to strong disagreement and a score of 7 indicates strong agreement (lower scores are indicative of elevated levels of ambiguity). For each area, a score from three to nine is considered low, a score from 10 to 15 is considered moderate, and a score from 16 to 21 is considered high. Job Role ambiguity total score for the nine items ranges from nine to 63. Regarding the overall degree of job Role ambiguity, nine to 27 is low, 28 to 45 is moderate, and 46 to 63 is high [34]. The Job Role Ambiguity revealed excellent reliability with an alpha of 0.907.

### Ethical considerations

The study received ethical approval from South Valley University’s Faculty of Nursing Research Ethics Committee (code: SVU-NUR-GER-21-5-11-2024). After that, the researchers submitted a request outlining the aim of the study to the SVU hospital authorities, who granted them approval. All participants involved in the study provided signed informed consent forms, confirming their willingness to participate. The researchers conveyed to the participants that their involvement was completely voluntary and that they had the right to withdraw at any moment without facing any consequences. The confidentiality of the data gathered was guaranteed. It strictly adhered to the ethical principles set forth in the Helsinki Declaration to maintain the integrity of the research.

### Validity and reliability

The WLEIS, GAD-7, and Job Role Ambiguity scales were translated into Arabic. Each item in each tool was translated from English to Arabic, and after finishing the translation, the Arabic language Consultant revised and edited all tools to confirm Arabic language simplicity and clarity. The validity was evaluated by five experts in the fields of Gerontological, Psychiatric, and Mental Health Nursing. The content’s Lawshe Content Validity Ratio, which was found to be more than 0.99, ensured the tools’ correctness, clarity, relevance, and applicability. Additionally, a pilot study with 25 intensive care unit nurses assessed the practicality and clarity of the research measures, confirming that no changes were necessary.

### Statistical analysis

The statistical package for social sciences, IBM SPSS-26, was used to examine the quantitative data from this study. Descriptive statistics, which included counts, percentages, averages, standard deviations, and minimum and maximum values, gave an overview of the data. Cronbach’s Alpha was used to assess the study tools’ reliability.

To examine the correlations between two quantitative variables, the Pearson correlation coefficient was employed. Additionally, using the SPSS-AMOS software, standardized regression weights, standard error (SE), and critical ratio (CR) were calculated to evaluate the direct and indirect relationships between anxiety and role ambiguity, which were mediated by emotional intelligence. Several goodness-of-fit statistics, including chi-square, the comparative fit index (CFI), the incremental fit index (IFI), and the root mean square error of approximation (RMSEA), were used to assess the model fit [10]. In this study, p-values were considered statistically significant if they fell within the range of 0.05 and 0.01.

## Results

Table 1 reveals socio-demographic and occupational data of critical care nurses. This table shows that 58.0% of nurses aged from 20 to less than 30 years, while 37.2% of them aged from 30 to less than 40 years, with a mean of 29.34 ± 5.432 years. As for sex, this table reveals that females constituted 79.2% of the studied nurses. Regarding marital status, 65.2% of the studied nurses were married, and 30.4% were single. In terms of education, 60% of the nurses studied were nursing technicians, compared to 31.6% of those with bachelor’s degrees. Regarding their shift, 53.2% of the studied nurses reported working alternatively between three shifts. Additionally, 95.2% and 70.0% of the studied nurses reported having less than 10 years of ICU and nursing profession experience, respectively.

**Table 1:** Distribution of the ICU nurses according to their sociodemographic and occupational data

Socio-demographic data	Frequency (*n* = 250)	Percent
Age		
♣ 20-	145	58.0
♣ 30-	93	37.2
♣ 40–50	12	4.8
Min- Max 20–50 Mean ± SD 29.34 ± 5.432		
**Sex**		
♣ Male	52	20.8
♣ Female	198	79.2
**Marital status**		
♣ Single	76	30.4
♣ Married	163	65.2
♣ Divorced	7	2.8
♣ Widow	3	1.2
♣ Separated	1	0.4
**Education**		
♣ Nursing diploma	18	7.2
♣ Nursing technician	150	60.0
♣ Bachelor	79	31.6
♣ Master	3	1.2
**Shift**		
♣ Morning only	44	17.6
♣ Evening only	1	0.4
♣ Long	65	26.0
♣ Night only	7	2.8
♣ Three shifts	133	53.2
**Profession experience**		
♣ < 10	175	70.0
♣ 10-	53	21.2
♣ 20-	21	8.4
♣ 30+	1	0.4
Min- Max 1–31 Mean ± SD 9.16 ± 6.184		
**ICU experience**		
♣ < 10	238	95.2
♣ 10-	10	4.0
♣ 20–30	2	0.8
Min- Max 1–24 Mean ± SD 2.86 ± 3.654		

Table 2 illustrates the distribution of the studied ICU nurses according to their emotional intelligence, which is explained under four subscales. Self-emotion appraisal is the first subscale; this table shows that 64.4% of the studied nurses reported having high self-emotion appraisal with a mean of 21.42 (4.131). The second and the third subscales are emotion regulation and use of emotion; it is noticed from this table that the studied nurses had high emotion regulation with a mean of 21.94 (3.541) and a mean of 23.05 (3.67) use of emotion. On the other hand, the fourth emotional intelligence subscale is others’ emotion appraisal, in which the studied nurses reported having a mean of 20.30 (3.967). Regarding emotional intelligence, this table reveals that 72.4% of the nurses studied had high emotional intelligence.

**Table 2:** Distribution of the ICU nurses according to their emotional intelligence

Emotional Intelligence	Frequency(*n* = 250)	Percent	Min- Max	Mean (SD)
Self-emotion appraisal				
♣ Low	9	3.6	4–28	21.42 (4.131)
♣ Moderate	80	32.0		
♣ High	161	64.4		
**Emotion regulation**				
♣ Low	4	1.6	10–28	21.94 (3.541)
♣ Moderate	73	29.2		
♣ High	173	69.2		
**Use of Emotion**				
♣ Low	4	1.6	8–28	23.05 (3.671)
♣ Moderate	42	16.8		
♣ High	204	81.6		
**Others emotion appraisal**				
♣ Low	8	3.2	12–28	20.30 (3.967)
♣ Moderate	117	46.8		
♣ High	125	50.0		
**Total Emotional Intelligence**				
♣ Moderate	69	27.6	55–112	86.71(11.217)
♣ High	181	72.4		

Table 3 reveals the distribution of the studied ICU nurses according to their reported anxiety level. This table explains that 46.4% and 22.4% of the studied nurses reported having mild and minimal anxiety levels. On the other hand, moderate and severe anxiety levels were prevalent among 21.6% and 9.6% of the studied ICU nurses.

**Table 3:** Distribution of the ICU nurses according to their reported anxiety level

Anxiety	Frequency (*n* = 250)	Percent	Min- Max	Mean (SD)
♣ Minimal Anxiety	56	22.4	0–21	7.98(4.558)
♣ Mild Anxiety	116	46.4		
♣ Moderate Anxiety	54	21.6		
♣ Severe Anxiety	24	9.6		

Table 4 describes how the ICU nurses in the study were distributed based on their role ambiguity. According to this table, 80.4% of the studied nurses reported having mild work method ambiguity. On the other hand, 79.6% and 70.4% of the studied ICU nurses reported low scheduling and performance ambiguity. Regarding total role ambiguity, 78.8% of the studied ICU nurses reported having a low level, with a mean of 51.61 (7.101).

**Table 4:** Distribution of the ICU nurses according to their reported role ambiguity

Role ambiguity	Frequency (*n* = 250)	Percent	Min- Max	Mean (SD)
Work method ambiguity				
♣ Low	201	80.4	6–21	17.49 (2.601)
♣ Moderate	42	16.8		
♣ High	7	2.8		
**Scheduling ambiguity**				
♣ Low	199	79.6	6–21	17.33(2.681)
♣ Moderate	43	17.2		
♣ High	8	3.2		
**Performance ambiguity**				
♣ Low	176	70.4	6–21	16.74 (2.948)
♣ Moderate	60	24.0		
♣ High	14	5.6		
**Total Role ambiguity**				
♣ Low	197	78.8	18–63	51.61 (7.101)
♣ Moderate	40	16.0		
♣ High	13	5.2		

Table 5 presents the correlation between the studied ICU nurses’ emotional intelligence, anxiety, and role ambiguity. This table shows that there is a statistically significant negative relationship found between the studied ICU nurses’ emotional intelligence and anxiety level (*r*=-0.366, *P* = 0.000). Furthermore, it is noticed from this table that a statistically significant negative relationship was found between the studied ICU nurses’ anxiety level and role ambiguity (as a higher score in the role ambiguity scale reflects less job ambiguity) (*r*=-0.327, and *P* = 0.000). Moreover, a statistically significant positive relationship was found between the studied ICU nurses’ emotional intelligence and role ambiguity (*r*=-0.630, *P* = 0.000).

**Table 5:** Correlation between the nurses’ emotional intelligence, anxiety, and role ambiguity

Correlations				
		EI	Anxiety	Role ambiguity
EI	Pearson Correlation		− 0.366^**^	0.630^**^
	Sig. (2-tailed)		0.000	0.000
Anxiety	Pearson Correlation			− 0.327^**^
	Sig. (2-tailed)			0.000

**Correlation is significant at the 0.01 level (2-tailed)

Table 6 illustrates a multiple linear regression between the nurses’ emotional intelligence, anxiety, and role ambiguity. It is noticed from this table that the regression model yielded a value of 8.765 (*P* = 0.024), reflecting a statistically significant relationship. Furthermore, this table shows that both emotional intelligence and role ambiguity impact ICU nurses’ anxiety levels, explaining 10.6% and 12.4% of the variance, respectively. Additionally, the ANOVA results confirm the model validity as (F = 17.633, *P* = 0.000). The model equation can help predict the ICU nurses’ anxiety level, considering their emotional intelligence and role ambiguity. The initial nurses’ anxiety level is 21.437, which decreases by 0.082 with each increase in emotional intelligence and by 0.126 with each rise in role ambiguity.

**Table 6:** Linear regression between the nurses’ emotional intelligence, anxiety, and role ambiguity

Coefficients ^a^										
		Unstandardized Coefficients		Standardized Coefficients	t	Sig.	ANOVA		*R* Square	*R* Square Change
Model		B	Std. Error	Beta			F	Sig.		
1	(Constant)	19.477	2.222		8.765	0.000	28.042	.000^b^	10.6%	10.6%
	EI	− 0.134	0.025	− 0.326	-5.296	0.000				
2	(Constant)	21.437	2.325		9.222	0.000	17.663	.000^c^	13.1%	
	EI	− 0.082	0.032	− 0.199	-2.541	0.012				10.6%
	Role ambiguity	− 0.126	0.049	− 0.201	-2.572	0.011				2.4%

a. Dependent Variable: Anxiety

b. Predictors: (Constant), EI

c. Predictors: (Constant), EI, Role ambiguity

Anxiety = 21.437 − 0.082* emotional intelligence − 0.126* role ambiguity

**Fig. 2:**
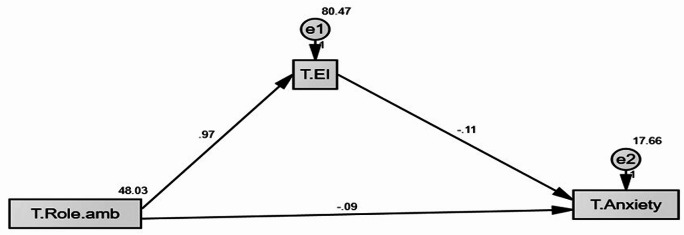
Regression analysis model

As displayed in Table 7; Fig. 2, the direct and indirect effects of emotional intelligence on the anxiety level and work ambiguity of intensive care unit nurses were evaluated using Amos-SPSS: standard error, critical ratio, and standard regression weights. The parameters for the model fit were (X2/df = 0.030/1.00, CFI = 1.000; IFI = 1.00; RMSEA = 0.000). The research variables provide rigorous estimations of a good root and a perfect comparative fit index, indicating a square approximation error.

**Table 7:** A path analysis of direct and indirect effects of nurses’ role ambiguity on anxiety mediated by emotional intelligence

Variable 1	Direction	Variable 2	Standardized Regression Weights		S.E.	C.*R*.	*P*	Significance
EI	<---	Role ambiguity	0.966	0.082		11.780	***	Significant
Anxiety	<---	EI	− 0.115	0.030		-3.861	***	Significant
Anxiety	<---	Role ambiguity	− 0.092	0.048		-1.924	0.054	Not Significant

Model X2; significance 0.030; 0.000

Model fit parameters CFI; IFI; RMSEA (1.00;1.00;0.000)

CFI: Comparative Fit Index, IFI: Incremental Fit Index, RMSEA: Root Mean Square Error of Approximation

Table 8 presents the results of a bootstrapped regression weights mediation analysis in which role ambiguity influences anxiety through emotional intelligence. Role ambiguity and emotional intelligence are found to be strongly and statistically significantly correlated (Estimate = 0.966, *p* = 0.004). Emotional intelligence, in turn, predicts anxiety considerably and negatively (Estimate = -0.115, *p* = 0.004), indicating that nurses who have higher EI have lower anxiety levels. There was no statistically significant direct relationship between role ambiguity and anxiety (Estimate − 0.092, *p* = 0.120), suggesting that role ambiguity predominantly has an indirect influence on anxiety that is mediated by emotional intelligence.

**Table 8:** Regression weights bootstrapping results of direct and indirect effects of nurses’ role ambiguity on anxiety mediated by emotional intelligence

Parameter			Estimate		Lower	Upper	*P*	SE	SE-SE	Mean	Bias	SE-Bias
EI	<--	Role ambiguity		0.966	0.794	1.155	0.004	0.093	0.003	0.966	0.000	0.004
Anxiety	<--	EI		− 0.115	− 0.183	− 0.039	0.004	0.038	0.001	− 0.114	0.000	0.002
Anxiety	<--	Role ambiguity		− 0.092	− 0.201	0.027	0.120	0.058	0.002	− 0.093	0.000	0.003

Table 9 explores the relationship between nurses’ sociodemographic and occupational characteristics and their levels of emotional intelligence, anxiety, and role ambiguity. The results reveal that gender did not significantly influence EI, anxiety, or role ambiguity, as indicated by non-significant p-values (*p* > 0.05) across all measures. Age, however, showed a significant relationship with anxiety (*p* = 0.017), where older nurses reported lower levels of anxiety. On the other hand, no significant age-related differences were found for EI or role ambiguity. Regarding marital status, this table shows that nurses’ marital status has no significant impact on any of the variables, either EI, role ambiguity, or anxiety level. Additionally, educational level showed significant associations with both emotional intelligence (*p* = 0.026) and anxiety (*p* = 0.003), with nurses holding diplomas demonstrating the highest EI and lowest anxiety. However, role ambiguity did not significantly differ across educational levels. As for professional experience, anxiety levels significantly differed by years of experience (*p* < 0.001), with more experienced nurses reporting lower anxiety levels. However, years of professional experience were not significantly associated with EI or role ambiguity. Similarly, no statistically significant differences were observed in EI, anxiety, or role ambiguity concerning years of ICU experience or shift type.

**Table 9:** Relationship between nurses’ Socio-Demographic and occupational data, their emotional intelligence, anxiety, and role ambiguity

Nurses’ Sociodemographic Data	No	Emotional Intelligence		Anxiety		Role ambiguity	
		Mean ± SD	Test of sig.	Mean ± SD	Test of sig.	Mean ± SD	Test of sig.
Gender							
♣ Males	52	85.79 ± 13.075	t = 0.664	7.52 ± 4.123	t=-0.811	50.58 ± 8.844	t=-1.159
♣ Females	198	86.95 ± 10.700	*p* = 0.508	8.10 ± 4.668	*p* = 0.418	51.89 ± 6.561	*p* = 0.248
Age							
♣ 20-	145	86.32 ± 11.339	F = 0.262	8.66 ± 4.731	F = 4.136	51.96 ± 6.448	F = 0.872
♣ 30-	93	87.12 ± 10.745	*P* = 0.770	7.14 ± 3.903	*p* = 0.017*	50.88 ± 7.903	*p* = 0.420
♣ 40–50	12	88.25 ± 13.903		6.25 ± 5.895		53.00 ± 8.158	
Marital status							
♣ Single	76	87.42 ± 11.911		8.71 ± 5.228		7.98 ± 7.591	
♣ Married	163	86.59 ± 11.029	F = 0.521	7.60 ± 4.262	F = 0.825	50.63 ± 6.953	F = 1.095
♣ Divorced	7	84.86 ± 9.771	*p* = 0.720	8.57 ± 4.117	*p* = 0.510	52.16 ± 5.314	*p* = 0.360
♣ Widow	3	78.67 ± 8.505		8.67 ± 2.309		51.29 ± 3.512	
♣ Separated	1	89.00 ± 0.00		7.00 ± 0.00		46.33 ± 0.00	
Level education							
♣ Nursing Diploma	18	91.94 ± 10.230	F = 3.130	4.83 ± 4.668	F = 4.729	53.12 ± 4.285	F = 1.051
♣ Technical Nursing Institute	150	87.35 ± 11.567	*P* = 0.026*	7.99 ± 4.501	*P* = 0.003*	52.01 ± 6.925	*P* = 0.371
♣ Bachelor of Nursing	79	84.11 ± 10.219		8.46 ± 4.239		50.59 ± 7.456	
♣ Master	3	91.33 ± 12.583		13.33 ± 7.095		49.00 ± 17.059	
Years of professional experience							
♣ < 10	175	85.90 ± 11.355	F = 1.811	8.73 ± 4.645	F = 7.214	51.38 ± 7.021	F = 0.578
♣ 10-	53	87.40 ± 10.113	*P* = 0.146	6.66 ± 3.293	*P* = 0.000*	51.57 ± 7.320	*P* = 0.630
♣ 20-	21	91.76 ± 12.037		4.81 ± 4.676		53.52 ± 7.441	
♣ 30–40	1	86.00 ± 0.00		4.00 ± 0.00		53.00 ± 0.00	
Years of ICU experience							
♣ < 10	238	86.79 ± 11.128	F = 0.562	8.01 ± 4.513	F = 1.212	51.81 ± 6.753	F = 2.456
♣ 10-	10	83.80 ± 13.998	*P* = 0.571	8.20 ± 5.750	*P* = 0.299	46.80 ± 12.630	*P* = 0.088
♣ 20–30	2	92.00 ± 8.485		3.00 ± 0.00		53.00 ± 2.828	
Shift							
♣ Morning only	44	89.30 ± 11.533		7.61 ± 4.794		53.05 ± 6.363	
♣ Evening only	1	95.00 ± 0.000		5.00 ± 0.00		60.00 ± 0.00	
♣ Morning &Evening	65	87.43 ± 9.472		8.25 ± 4.744		51.49 ± 6.770	
♣ Night only	7	85.29 ± 7.017	F = 1.200	9.71 ± 6.448	F = 0.494	47.00 ± 6.753	F = 1.470
♣ Three shifts	133	85.51 ± 11.988	*P* = 0.311	7.89 ± 4.312	*P* = 0.740	51.35 ± 7.444	*P* = 0.212

## Discussion

Emotional intelligence (EI) plays a crucial role in the well-being of nurses caring for critically ill geriatric patients, particularly in mitigating anxiety and role ambiguity. In high-stress environments like intensive care units, nurses often face significant emotional and psychological challenges due to the complexities of patient care and the inherent uncertainties of their roles. The current study aimed to investigate the role of emotional intelligence as a mediator between anxiety and role ambiguity among nurses caring for critically ill geriatric patients.

The current study findings provide a comprehensive overview of nurses’ emotional intelligence (EI) across four key dimensions: self-emotion appraisal, emotion regulation, use of emotion, and others’ emotion appraisal. These dimensions align with established EI frameworks and offer valuable insights into the emotional competencies of nursing professionals. Self-emotion appraisal, which involves recognizing and understanding one’s emotions, shows that most nurses score in the high range [35]. The non-significant direct path from role ambiguity to anxiety (highlights a complex relationship that may warrant further exploration. This finding contradicts prior literature, suggesting that emotional intelligence may play a more significant role in buffering the effects of role ambiguity than previously understood [36].

This ability is crucial for nurses, as it forms the foundation for effective emotional management in high-stress clinical environments. Research has shown that nurses with strong self-emotion appraisal are better equipped to handle the emotional demands of their profession, leading to improved job satisfaction and reduced burnout [37].

Emotion regulation demonstrates even higher proficiency among nurses. This skill is vital in nursing, where maintaining professional composure in emotionally charged situations is essential. A previous study has indicated that nurses with superior emotion regulation skills are more resilient to stress and can better provide high-quality patient care [38]. Moreover, the emotion dimension, which involves harnessing emotions to facilitate performance, shows the highest percentage of nurses in the high category. This denotes that most nurses can use emotions to enhance their clinical abilities and decision-making processes. Supporting the current result, it was previously found that using emotions can lead to improved critical thinking and clinical performance among nurses [39].

Furthermore, others’ emotion appraisal shows a more balanced distribution between moderate and high scores. This skill is fundamental for empathetic patient care and effective communication with patients, families, and colleagues. Studies have demonstrated that nurses with strong abilities in this area tend to have better therapeutic relationships with patients and improved teamwork with colleagues [17, 40, 41]. The overall emotional intelligence scores indicate that a significant majority of nurses demonstrate high total EI. This is encouraging, as high EI has been associated with numerous positive outcomes in nursing practice, including enhanced quality of care, improved patient satisfaction, and better stress management.

The data indicate a significant prevalence of anxiety among nurses, with varying degrees of severity ranging from minimal to severe anxiety. The findings reveal that a substantial portion of nurses experience mild anxiety, which is the most frequently reported level. This shows that many nurses are grappling with anxiety that, while not classified as severe, may still impact their overall well-being and job performance. Mild anxiety can lead to increased stress and may affect decision-making and interpersonal relationships within the healthcare team. Research has shown that anxiety among healthcare workers can lead to burnout and decreased job satisfaction, ultimately affecting patient care quality [42].

The presence of moderate and severe anxiety levels among a notable percentage of nurses is particularly concerning. Moderate anxiety can hinder a nurse’s ability to perform effectively, while severe anxiety may lead to significant impairment in daily functioning. Studies have indicated that high levels of anxiety in healthcare professionals can result in increased absenteeism, reduced productivity, and a higher likelihood of making errors in patient care [43]. The mental health of nurses is critical not only for their well-being but also for the safety and quality of care provided to patients. This is especially important in high-stress settings when prompt decision-making and concentrated attention are critical, such as in emergency rooms or intensive care units. Various factors contribute to anxiety among nurses. These may include challenging work environments, lack of human resources, ongoing changes in patient care delivery, and the emotional demands of the profession [44].

Moreover, the findings indicate that role ambiguity is often rooted in systemic issues such as poor leadership and unclear policies. Addressing these structural factors is essential for mitigating role ambiguity and its associated anxiety. Strong leadership, clear communication channels, and well-defined policies are vital in creating an environment where nurses understand their roles and responsibilities [36]. This organizational support is crucial for fostering a work environment that clarifies expectations and reduces uncertainty.

The findings show that many nurses experience low work method ambiguity regarding role ambiguity. This indicates they clearly understand the procedures and methods required in their roles. Clarity in work methods is crucial as it allows nurses to perform their tasks efficiently and confidently, reducing the likelihood of errors and enhancing patient care quality. Research has demonstrated that role clarity is associated with lower stress levels and higher job satisfaction among healthcare professionals [45].

Similarly, the data on scheduling ambiguity reveals that most nurses report low levels of uncertainty regarding their work schedules. This clarity is vital for maintaining work-life balance and ensuring nurses can effectively manage their personal and professional responsibilities. It was previously reported that predictable work schedules contribute to job satisfaction and reduce burnout, which is particularly important in the demanding field of nursing [46]. While most nurses report low-performance ambiguity, there is still a notable percentage experiencing moderate to high levels of uncertainty regarding performance expectations. This aspect is critical, as unclear performance expectations can lead to anxiety and decreased job performance. A previous study indicates that role ambiguity can negatively impact job satisfaction and increase the likelihood of burnout among nurses [47].

Regarding the relationship between the study variables, the findings illustrate the negative correlation between emotional intelligence and anxiety, indicating that higher levels of emotional intelligence are associated with lower anxiety levels among nurses. This finding aligns with existing research demonstrating how emotional intelligence equips individuals with the skills to effectively manage stress and emotional challenges [48]. Nurses with higher emotional intelligence are better able to navigate the emotional demands of their roles, leading to reduced feelings of anxiety [49]. Studies have shown that emotional intelligence facilitates adaptive coping strategies, significantly lowering anxiety levels in high-pressure environments, such as healthcare settings [49, 50].

The positive correlation between emotional intelligence and role ambiguity (where higher scores indicate less ambiguity) indicates that higher emotional intelligence is associated with lower levels of role ambiguity. This relationship is critical, as it points out that emotionally intelligent nurses are more capable of understanding and clarifying their roles and responsibilities. By effectively managing their emotions and empathizing with colleagues, these nurses can foster better communication and teamwork, reducing uncertainty regarding job roles. Research supports the notion that emotional intelligence enhances role clarity, improving job satisfaction and performance among healthcare professionals [50].

The negative correlation between anxiety and role ambiguity further emphasizes the implications of these findings. As anxiety levels decrease, role ambiguity also diminishes, denoting that when nurses feel more confident and less anxious, they can better define and understand their roles. This relationship is particularly relevant in nursing, where role ambiguity can increase stress and job dissatisfaction. Studies have indicated that reducing role ambiguity through clear communication and defined expectations can alleviate anxiety, promoting a healthier work environment for nurses [1, 47].

The linear regression analysis reveals that both emotional intelligence and role ambiguity are important predictors of anxiety levels among nurses, with emotional intelligence showing a notable impact. The regression analysis demonstrates that emotional intelligence has a significant negative relationship with anxiety. This shows that as emotional intelligence increases, anxiety levels decrease. The unstandardized coefficient for emotional intelligence indicates that anxiety decreases by a certain amount for each unit increase in emotional intelligence. This finding is consistent with existing literature, which posits that higher emotional intelligence enables nurses to manage stress and emotional challenges better, thereby reducing anxiety levels [51]. Emotional intelligence equips nurses with the skills to navigate the emotional demands of their roles, leading to improved mental health outcomes.

In addition to emotional intelligence, role ambiguity also significantly predicts anxiety levels. The regression results indicate that higher levels of role ambiguity are associated with increased anxiety among nurses. This relationship underscores the importance of clear role definitions and expectations in nursing. When nurses experience role ambiguity, they may feel uncertain about their responsibilities, leading to heightened anxiety. Research has shown that role clarity is essential for job satisfaction and can mitigate stress and anxiety in healthcare settings [52].

Moreover, the findings indicate that increased role ambiguity correlates with heightened anxiety levels among nurses, while emotional intelligence acts as a buffer, alleviating this anxiety. This observation supports existing research that underscores the vital role of emotional intelligence in managing stress and anxiety in demanding healthcare environments [51]. Studies have indicated that nurses possessing higher emotional intelligence are more capable of handling the emotional challenges of their roles, resulting in lower anxiety and greater job satisfaction [51, 52]. In contrast, role ambiguity has been associated with heightened stress and burnout, as unclear job expectations can intensify feelings of inadequacy and anxiety among healthcare workers [47].

Furthermore, the results highlight differences in emotional intelligence and anxiety levels across various demographic groups, suggesting that factors like age, education, and years of experience impact these variables. For instance, younger and less experienced nurses tend to report higher anxiety levels, aligning with findings that indicate novice nurses face greater difficulties adjusting to their roles [53]. Moreover, educational attainment seems to influence emotional intelligence and anxiety, with those holding diplomas displaying different profiles compared to their counterparts with advanced degrees.

### Limitations

This study has certain limitations that should be acknowledged. Firstly, the research was conducted in a single hospital setting within South Valley University, which may limit the generalizability of the findings to other healthcare institutions or regions. While adequate for the statistical analyses, the sample size may not fully represent the diversity of the nursing workforce, particularly in terms of experience levels and specializations. Moreover, the study’s cross-sectional design restricts the ability to draw causal inferences regarding the relationships between emotional intelligence, anxiety, and role ambiguity. Additionally, the regression model accounted for only 13.1% of the variance in anxiety, indicating that other influential factors may not have been captured. Future research should consider incorporating additional variables such as workload, organizational support, and other contextual or psychological factors to provide a more comprehensive understanding of anxiety in this setting. Lastly, self-reported measures, such as the Wong and Law Emotional Intelligence Scale (WLEIS), GAD-7, and Job Role Ambiguity assessment, may be subject to response biases, including social desirability bias, which could affect the accuracy of the data collected.

### Conclusion

The study underscores the significant role of emotional intelligence in mitigating anxiety and role ambiguity among nurses caring for critically ill geriatric patients. The findings indicate that nurses with higher emotional intelligence are better equipped to manage stress and emotional challenges, leading to reduced anxiety levels and more explicit role definitions. This relationship highlights the importance of fostering emotional intelligence within nursing, particularly in high-stress environments like intensive care units. The results also emphasize the need for healthcare organizations to address role ambiguity to enhance nurses’ job satisfaction and overall well-being. By promoting emotional intelligence and clarifying role expectations, healthcare systems can contribute to improved mental health outcomes for nurses, which positively influence patient care quality.

### Recommendations

Based on the findings of this study, several recommendations can be made. Firstly, healthcare organizations should implement structured training programs to enhance emotional intelligence (EI) among nursing staff. These programs should span 4–6 weeks, with weekly sessions of 1–2 h each. The curriculum should focus on developing essential skills in self-emotion appraisal, emotion regulation, and empathetic communication. Additionally, incorporating evidence-based practices such as mindfulness techniques and effective communication workshops will further equip nurses to navigate the emotional demands of their roles more effectively.

Secondly, it is crucial to address systemic issues contributing to role ambiguity within nursing teams. Organizations should establish clear job descriptions, expectations, and communication channels to reduce confusion and enhance role clarity. Regular team meetings and feedback sessions can facilitate better understanding and collaboration among staff, thereby minimizing uncertainty and improving job satisfaction. This approach acknowledges that role ambiguity is often rooted in poor leadership and unclear policies, which must be addressed to create a supportive work environment.

Additionally, organizations should consider providing comprehensive mental health support services, including counseling and tailored stress management workshops. These services will help nurses cope with anxiety and stress, particularly in high-pressure settings like intensive care units. Supporting nurses’ mental health is essential for fostering a healthier work environment and ensuring that they can perform their duties effectively.

Finally, future research should explore the long-term effects of emotional intelligence training on nurse well-being and patient care outcomes. Investigating the role of other potential mediators or moderators in the relationship between emotional intelligence, anxiety, and role ambiguity is also vital. This includes examining how structural factors influence these dynamics, which could lead to more effective interventions in the future. By promoting structured emotional intelligence training, addressing systemic issues related to role ambiguity, and providing robust mental health support, healthcare systems can significantly enhance the well-being of nurses, ultimately leading to improved quality of patient care.

## Data Availability

The datasets used or analyzed in this study are available from the corresponding author upon request.
